# Atrial electrical alterations with intact cardiac structure and contractile function in a mouse model of an HCM-linked *ACTN2* variant

**DOI:** 10.1016/j.jmccpl.2025.100455

**Published:** 2025-05-17

**Authors:** Maya Noureddine, Sophie Broadway-Stringer, Christopher O'Shea, Bethany A.I. Jones, Abbie Hayes, Chris Denning, Siobhan Loughna, Fiyaz Mohammed, Davor Pavlovic, Katja Gehmlich

**Affiliations:** a Department of Cardiovascular Sciences, School of Medical Sciences, College of Medicine and Health, University of Birmingham, Birmingham, UK; b Division of Biomedical Sciences, Warwick Medical School, Clinical Sciences Research Laboratory, Coventry, UK; c Biodiscovery Institute, University of Nottingham, Nottingham, UK; d School of Life Sciences, Faculty of Medicine and Health Sciences, University of Nottingham, Nottingham, UK; e Department of Immunology and Immunotherapy, School of Infection, Inflammation and Immunology, College of Medicine and Health, University of Birmingham, Birmingham, UK; f Division of Cardiovascular Medicine, Radcliffe Department of Medicine and British Heart Foundation Centre of Research Excellence Oxford, University of Oxford, Oxford, UK

**Keywords:** Alpha-actinin-2 (ACTN2), Hypertrophic cardiomyopathy (HCM), Pathogenic variants, Atrial electrophysiology, Cardiac remodelling

## Abstract

**Background:**

Missense variants of *Z*-disk protein, alpha-actinin-2 (ACTN2), have been linked to hypertrophic cardiomyopathy (HCM). A novel *ACTN2* missense variant, M228T, was identified in family members presenting with HCM and/or atrial arrhythmias. Embryonic lethality was previously shown in mice expressing this variant homozygously, whereas heterozygous (Het) expression did not manifest an overt HCM phenotype. Importantly, the atrial arrhythmias observed in the identified family have not been explored in the context of M228T, despite many patients exhibiting electrical abnormalities prior to the clinical onset of HCM.

**Methods:**

Six-month-old Het M228T and wild-type (WT) mice were used to evaluate electrophysiological properties using electrocardiography (ECG) and atrial optical mapping. Echocardiography and strain analysis were employed to assess cardiac structure and function.

**Results:**

Het mice exhibited a prolongation in action potential duration and depolarisation time at 30, 50, and 70 % repolarisation in both the left and right atria. No significant alterations in atrial conduction velocity were observed. No changes in atrial ECG parameters were detected. Het mice displayed no evidence of structural remodelling, nor were there any changes in systolic parameters or overt diastolic dysfunction, as assessed by conventional echocardiography and strain analysis. Signs of contractile dyssynchrony were present, specifically at the apex relative to WT controls.

**Conclusion:**

The Het M228T mouse model demonstrated atrial electrical alterations that occurred independently of any overt cardiac structural or functional remodelling. These findings may support the causative role for atrial electric phenotypes identified in a subset of patients carrying the variant.

## Highlights

• Mouse model of *ACTN2* HCM-associated M228T variant displays atrial electrical alterations.
• Prolonged atrial action potential duration in Het mice may indicate an arrhythmogenic mechanism.
• Het mice demonstrate apical left ventricular contractile dyssynchrony without structural remodelling or impairment.
• Electrical alterations are independent of structural remodelling.

## Graphical abstract

**Figure:**
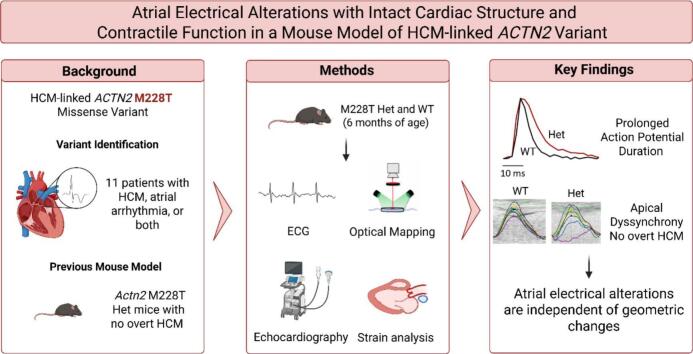

## 1 Introduction

Inherited cardiomyopathies represent a group of genetic diseases that significantly impact myocardial function and structure. Among these, hypertrophic cardiomyopathy (HCM) is the most prevalent type [1], characterised primarily by increased left ventricular (LV) wall thickness and diastolic dysfunction. Patients with HCM frequently exhibit electrophysiological abnormalities, including atrial fibrillation (AFib) [2]. It has been proposed that the heightened risk of arrhythmias in these patients is associated with adverse structural changes such as atrial remodelling and dysfunction [3,4]. This structural remodelling involves alterations in the sarcomeric contractile apparatus, thereby contributing to disease progression.

**Fig. 1:**
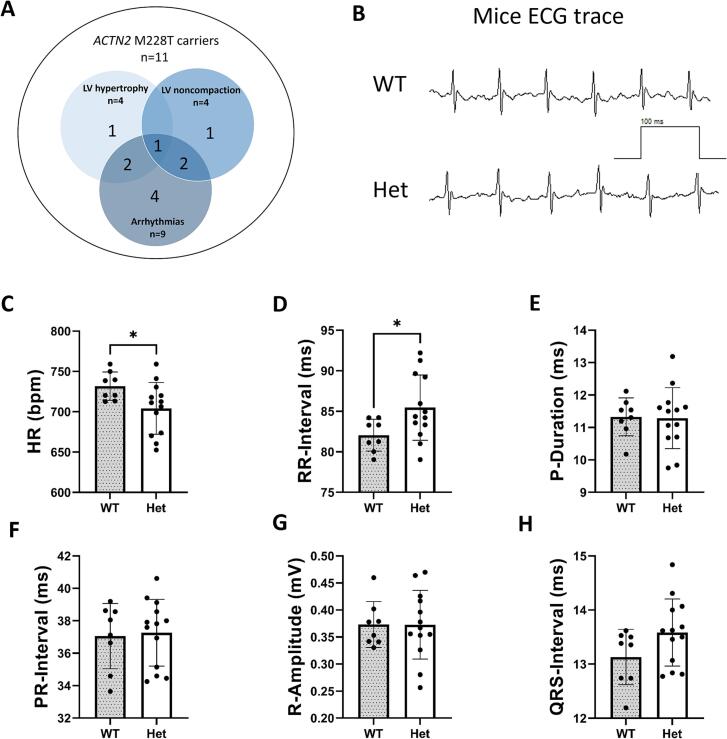
Venn diagram of *ACTN2* M228T carriers' phenotype and ECG analysis of wildtype (WT) and *Actn2* M228T heterozygous (Het) at six months of age reveals alterations in heart rate (HR) and RR-interval. (A) Venn diagram illustrating the phenotypes among 11 carriers of the *ACTN2* M228T variant, with four exhibiting LV hypertrophy, four displaying LV noncompaction, and nine presenting with arrhythmias. (B) Representative ECG traces for WT and Het groups. (C, D, E) Analysis of HR and RR-interval, and P-wave. (F, G, H) Analysis of PR-interval, R-amplitude, and QRS-interval. Data were normally distributed and unpaired *t*-tests were used. Values are presented as mean ± SD *p < 0.05; (WT *n* = 8, Het *n* = 13). Abbreviations: bpm, beats per minute.

Alpha-actinin-2 (ACTN2) is a key protein located within the *Z*-disk that constitutes the lateral boundaries of the sarcomere. ACTN2 is essential for stabilising sarcomeres through its cross-linking activity with actin thin filaments [5]. Several studies have identified various *ACTN2* missense variants that are associated with different types of cardiomyopathies, with a significant majority linked to HCM [6]. The HCM-linked *ACTN2* variant M228T (p.Met228Thr) was identified in 11 patients from a large four-generation family [7]. Among these patients, eight patients displayed autosomal dominant cardiomyopathic features, including LV hypertrophy and/or LV noncompaction (Fig. 1A). Moreover, nine of the 11 identified individuals exhibited atrial electrical abnormalities, with each patient experiencing one to four complications. The report documented two cases of early onset AFib, two cases of atrial flutter, six instances of first-degree or complete atrioventricular block, three of premature atrial contractions, three of paroxysmal supraventricular tachycardia, and one instance of sinoatrial block. Interestingly, four of the nine patients with atrial arrhythmias did not show symptoms of HCM (Fig. 1A). For example, the index patient experienced paroxysmal AFib at age 30 but was only diagnosed with HCM in his fifties [7].

A recent study by our group investigated the impact of the *ACTN2* M228T variant using a mouse model, which demonstrated embryonic lethality in homozygous offspring, highlighting the deleterious consequences of the variant on ACTN2 function, while heterozygous (Het) offspring did not display an overt HCM phenotype at nine months of age [8]. Notably, no electrical phenotypes were examined in this mouse model, despite the presence of cardiac electrical abnormalities among the identified patients. The current study focusses on atrial electrophysiological changes associated with the *ACTN2* M228T variant, to gain a deeper understanding of the electrical phenotype observed in the original family. Additionally, our work aims to assess potential underlying structural and functional changes in the hearts of mutant mice using strain analysis. The findings from this research will pave the way for future investigations into how variants of this *Z*-disk protein, and others, may contribute to the development of arrhythmias.

## 2 Materials and methods

### 2.1 Ethical statement

Animal studies were performed in accordance with the ethical standards outlined in the 1964 Declaration of Helsinki and its subsequent amendments. All experimental procedures adhered to the UK Home Office guidelines under the project licences PDCE16CB0 and PP6577680. Mice were provided with food and water *ad libitum* and maintained on a cycle of 12-hour light/dark cycle. Animals of both sexes were used at six months of age. All experiments and their corresponding offline analyses were blinded. Data from equal groups of male and female Het mice were compared to those from their wild-type (WT) littermates.

### 2.2 Generation of mouse model

The Het M228T missense variant in *Acnt2* was generated using CRISPR-Cas9-mediated homology-directed repair in C57BL/6J mice as described previously [8]. Het mice carrying the *Actn2* M228T variant were bred with WT C57BL/6J mice (Envigo, London, UK), and the expression of the *Actn2* M228T variant was previously confirmed by mass spectrometry [8].

### 2.3 Electrocardiography (ECG)

ECG was performed on conscious WT and Het *Actn2* M228T mice at six months of age, following previously established protocols [9]. Each mouse was gently placed into an ECG tunnel (EMKA Technologies) equipped with embedded ECG electrodes. The mice were allowed to acclimate to the tunnel before recording commenced using IOX2 recording software (EMKA Technologies, v2.9.4.34). The recordings were performed according to the manufacturer's instructions. Data were saved and analysed using ECGauto software (EMKA Technologies, v3.3.0.24). The following parameters were analysed: heart rate, RR-interval, PR-interval, P-duration, R-amplitude, and QRS-interval.

### 2.4 Cardiac optical mapping of mouse atria

Optical mapping of the atria from WT and Het *Actn2* M228T mice was conducted at six months of age, as previously described [10,11]. Briefly, the heart was extracted and mounted on a vertical Langendorff apparatus and retrogradely perfused with warmed Krebs solution (mM); 118 NaCL, 3.52 KCL, 0.83 MgSO_4_, 1.18 KH_2_PO_4_, 24.9 NaHCO_3_, 4.0 CaCl_2_ and 11 glucose. Warmed Krebs solution was mixed with the voltage-sensitive dye, Di-4-ANEPPS (5 mg/ml), and infused *via* aortic cannulation. The left and right atria were subsequently dissected and pinned into a 35 mm optical mapping rig and superfused with warmed circulating Krebs solution with mechanical uncoupler Blebbistatin (42.75 μM). Dye-loaded atria were excited using LED illumination at 530/50 nm and paced with bipolar platinum electrodes at pacing cycle lengths of 120, 100, and 80 ms. Data analysis was performed as previously described [12]. The following parameters were analysed: action potential duration (APD) at 30, 50 and 70 % repolarisation, depolarisation time, conduction velocity (CV), triangulation, normalised upstroke velocity (dF/dt) [13], and APD70 duration alternans analysis. A detailed methodology is available in the Supplementary Materials.

### 2.5 Tissue collection and tibia measurement

The heart was removed, divided into four chambers, and weighed separately (left and right atria; left (including septal wall) and right ventricles). The left and right lung lobes were harvested and weighed together. Mouse hind limbs were dissected and incubated overnight in 0.8 M NaOH solution (Sigma Aldrich) at room temperature to facilitate the digestion of the muscle tissue surrounding the tibia. Tibias were then incubated at 37 °C for 1 h to further digest the muscle tissue. The mouse patella and feet were removed to obtain accurate tibial measurements. Tibial length was measured using a digital calliper.

### 2.6 Immunofluorescence

Immunolabelling with Wheat Germ Agglutinin (WGA) Alexa Flour 488 (Thermofisher Scientific, Waltham, MA, USA, used 1:1000) was carried out on unfixed, un-permeabilised cryosections (7 um) of the right and left atrial tissues of the hearts as described [8].

### 2.7 Quantitative PCR (qPCR)

The mRNA isolation from the left and right atrial tissues, reverse transcriptase and quantitative PCR (qPCR) were performed as described [14]. The TaqMan probes (Applied Biosystems, Waltham, MA, USA) used are listed in Table S1.

### 2.8 Echocardiography

To assess cardiac function, echocardiography was performed on WT and Het *Acnt2* M228T mice at six months of age, as previously described [8]. A Vevo F2 ultrasound system and UHF57X transducer (Fujifilm VisualSonics) were used. M-mode and two-dimensional echocardiography (B-mode) images were acquired from both the parasternal long axis (PLAX) and short-axis (SAX) views. Pulsed-wave doppler images of mitral inflow were acquired. A detailed methodology is provided in the Supplementary Materials.

### 2.9 Speckle tracking echocardiography

Two-dimensional speckle tracking echocardiography and strain analysis (VevoLabs Strain 1.0 software) were performed on B-mode cine loops of the PLAX view of the heart from WT and Het *Actn2* M228T mice at six months of age. The software divided the left ventricular wall into six equal anatomical segments. The radial and longitudinal strain, strain rate, and time-to-peak (TTP) were averaged across the six segments over three consecutive cardiac cycles. A reverse-peak tool was employed to assess the strain, strain rate, and TTP during diastole. Maximal opposing wall delay (MOWD) was also calculated. A detailed methodology is provided in the Supplementary Materials.

### 2.10 Statistical analysis

Statistical analysis was performed using the GraphPad Prism software (version 9.0; GraphPad Inc., San Diego, CA, USA). All data are expressed as mean ± standard deviation (SD). Data were tested for normal distribution using the Shapiro-Wilk test. Unpaired *t*-tests were used for normally distributed data in two-group comparisons. For non-normally distributed data, the Mann-Whitney *U* test was used. Two-way ANOVA was applied to compare two groups across two or more categorical variables. One-way ANOVA was used for comparisons involving three or more groups in normally distributed data. For non-normally distributed data, a Kruskal-Wallis test was used. Statistical significance was set at *p* ≤ 0.05, with significance levels are indicated as follows: *p < 0.05, **p < 0.01, ***p < 0.001.

## 3 Results

### 3.1 Het Actn2 M288T mice display no changes in atrial electrophysiology using electrocardiography (ECG)

The cardiac electrical activity of *Actn2* M228T Het male and female mice was assessed using conscious ECG relative to that of their WT littermates at six months of age. This is equivalent to approximately 30 years in human age [15], the time point when the index patient with *ACTN2* M228T was diagnosed with AFib [7]. Representative ECG traces for both WT and Het mice are illustrated in Fig. 1B. ECG data analysis revealed that Het mice showed a significant reduction in heart rate accompanied by a corresponding increase in the RR-interval compared to the WT group (Fig. 1C, D). Notably, there were no significant differences observed in the P-duration, PR-interval, R-amplitude, or QRS-interval between Het mice and WT littermates (Fig. 1E, F, G, H).

### 3.2 Atria from Het mice show prolonged action potential duration and depolarisation time

An in-depth analysis utilising optical mapping of the left and right atria was performed. The atria were paced at cycle lengths of 120, 100, and 80 ms, which physiologically correspond to 500, 600, and 750 beats per minute (bpm), respectively. The action potential duration (APD) was measured at 30, 50, and 70 % repolarisation.

**Fig. 2:**
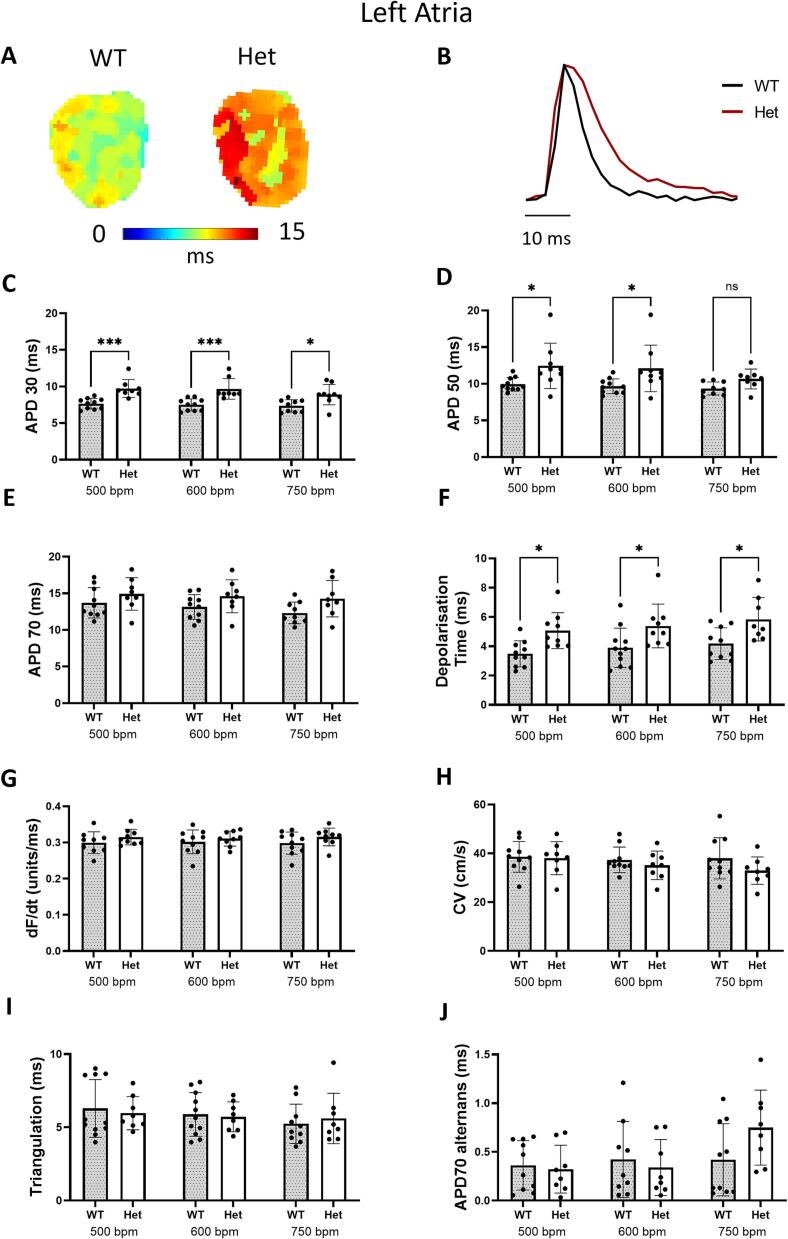
Optical mapping analysis of left atria from wildtype (WT) and *Actn2* M228T heterozygous (Het) at six months of age demonstrates atrial electrical alterations. (A) Representative heat maps at 50 % of repolarisation at 500 bpm for the left atria showing a delay in Het mice. (B) Representative action potential traces of left atria. (C, D, E) Analysis of action potential duration (APD) at 30, 50 and 70 % of repolarisation (APD 30, APD 50, and APD 70, respectively) showing an increase in Het mice. (F) Depolarisation time analysis of left atria shows significant increase. (G) Upstroke (dF/dt) in left atria show no significant changes. (H) Conduction velocity (CV) show no significant changes in left atria. (I) Triangulation derived from the difference between APD 70 and APD 30 in Het mice show no significant changes in left atria. (J) Alternans measurements show no significant differences in left atria. Two-way ANOVA was used to analyse data. Values are presented as mean ± SD *p < 0.05, ***p < 0.001. (WT, *n* = 10; Het, *n* = 9).

Representative heat duration depicting APD at 50 % repolarisation and at a pacing rate of 500 bpm, along with representative action potential traces for the left atria are presented in Fig. 2A, B. Statistical analysis of the APD in the left atria showed that Het mice exhibited a significant increase in APD at 30 % repolarisation compared to WT controls at all measured pacing rates (Fig. 2C). The APD at 50 % repolarisation was significantly increased at pacing rates of 500 and 600 bpm in the Het group, with no significant change in APD 70 (Fig. 2D, E). Interestingly, the depolarisation time was significantly elevated in the Het group at pacing rates of 500, 600, and 750 bpm (Fig. 2F). The analysis of upstroke (dF/dt) measurements in the left atria was also performed, but revealed no significant changes (Fig. 2G). No alterations in conduction velocity (CV) were detected (Fig. 2H). Triangulation, derived from the difference between APD 70 and APD 30 as described previously as a tool to predict arrhythmic outcomes [16,17], revealed no significant changes in the left atrium (Fig. 2I). Alternans analysis of duration showed no significant changes between the two genotypes (Fig. 2J).

Analysis of the right atria showed similar trends to that of the left atria, with a significant prolongation in APD 30 at pacing rate of 500 bpm (Fig. S1 C). APD at 50 % repolarisation was also elevated in the Het group at pacing rates of 500 and 600 bpm, with no significant changes in APD 70 (Fig. S1D, E). Depolarisation time in the right atria was also significantly increased at pacing rates 500 and 600 bpm in Het mice relative to WT mice (Fig. S1F). Analysis of dF/dt showed no significant changes (Fig. S1G). No significant changes in CV, triangulation or alternans in the right atria were observed (Fig. S1H, I, J).

### 3.3 Six-month-old M288T Het mice do not display an overt HCM phenotype

To determine whether the electrical changes observed in Het mice were associated with underlying cardiac structural remodelling, signs of cardiac hypertrophy were assessed. The analysis of whole heart weight and individual chamber weights normalised to tibia length (TL) revealed no significant differences between genotypes (Table 1A). Lung weight, an indicator of congestive heart failure, was not altered either when normalised to TL (Table 1A).

**Table 1:** Gravimetry and echocardiographic parameters of wildtype (WT) and *Actn2* M228T heterozygous (Het) at six months of age indicates no significant changes. (A) Gravimetry data showing no significant changes between the two groups. (B) Analysis of M-mode images in the PLAX view showing no significant changes. (C) Analysis of LA area and FAC using B-mode images in the PLAX and SAX view, respectively, showing no significant changes. Unpaired *t*-tests were used as data was normally distributed. Values are presented as mean ± SD. Abbreviations: BW, body weight; TL, tibia length; HW, heart weight; RA, right atrium; LA, left atrium; RV, right ventricle; LV, left ventricle; LW, lung weight; PLAX, parasternal long axis view; HR, heart rate; bpm, beats per minute; EF, ejection fraction; FS, fractional shortening; LV vol;d, left ventricular volume at end-diastole; LV vol;s, left ventricular volume at end systole; SAX, short axis view; FAC, endocardial fractional area change.

Parameter	WT (n = 14)	Het (n = 13)	*P*-value
A) Gravimetry			
BW (g)	28.6 ± 4.0	28.5 ± 4.3	0.66
BW/TL (g/mm)	1.65 ± 0.25	1.65 ± 0.21	0.68
HW/TL (mg/mm)	7.7 ± 1.0	8.4 ± 1.9	0.35
RA/TL (mg/mm)	0.18 ± 0.04	0.20 ± 0.09	0.59
LA/TL (mg/mm)	0.16 ± 0.05	0.17 ± 0.06	0.85
RV/TL (mg/mm)	1.01 ± 0.25	1.15 ± 0.34	0.28
LV + Septum/TL (mg/mm)	5.0 ± 0.8	5.4 ± 1.2	0.31
LW/TL (mg/mm)	9.4 ± 0.7	8.9 ± 0.8	0.19


Parameter	WT (n = 10)	Het (n = 11)	*P*-value
B) M-mode (PLAX)			

Age (weeks)	24.7 ± 0.45	24.8 ± 0.35	0.37
HR (bpm)	452 ± 42	430 ± 34	0.27
EF (%)	49.8 ± 13.8	50.3 ± 17.6	0.94
FS (%)	25.5 ± 8.7	26.1 ± 11.1	0.87
LV mass (mg)	161 ± 38	187 ± 76	0.33
LV vol;d (μL)	69.7 ± 10	75.8 ± 30	0.55
LV vol;s (μL)	36 ± 13.8	41 ± 28.2	0.80


Parameter	WT (n = 8)	Het (n = 8)	P-value
C) B-mode			

PLAX: LA area (mm^2^)	5.37 ± 0.8	5.83 ± 1.2	0.40
SAX: FAC (%)	41.9 ± 10.1	39.4 ± 7.5	0.57

To further assess for evidence of structural remodelling in the atria, mRNA expression analysis of the markers of fibrosis (*Col1a* and *Lox*) and hypertrophy (*Ankrd1* and *Fhl1*) was performed. It showed no significant changes in the left or right atria (Fig. S2A, B). Immunofluorescence microscopy was performed on the left and right atrial tissue using Wheat Germ Agglutinin (WGA) staining, showing no evidence of cellular hypertrophy in the Het mice (Fig. S2C).

**Fig. 3:**
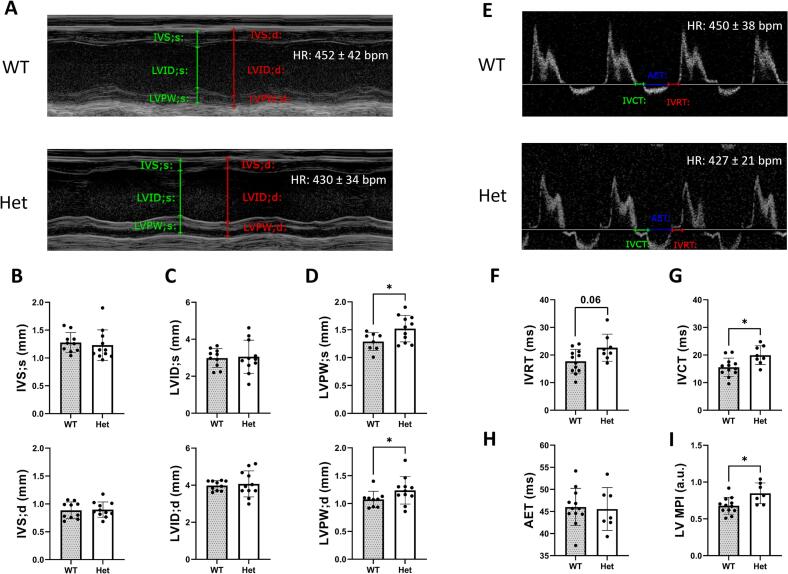
*Actn2* M228T Heterozygous (Het) mice show no overt structural or functional changes at six months of age when compared to their wildtype (WT) littermates. (A) Representative M-mode images in PLAX view showing tracing of IVS, LVID, and LVPW at systole (s) and diastole (d) in both WT and Het mice. (B, C) Het mice show no significant changes in IVS;s, IVS;d, LVID;s, and LVID;d. (D) Het mice show significant increase in LVPW;s and LVPW;d; (WT, n = 10; Het, n = 11, three consecutive readings are selected). (E) Representative image of pulse-wave doppler images on mitral inflow showing tracing of IVCT, AET, and IVRT in both WT and Het mice. (F) Het mice show a tendency for increase in IVRT. (G) Het mice show significant increase in IVCT. (H) No change in AET in Het mice. (I) Het mice show significant increase in LV MPI. Unpaired t-tests were used as data was normally distributed. Values are presented as mean ± SD *p < 0.05 (B-D: WT, n = 12; Het, n = 9, F-I: WT, *n* = 12; Het, *n* = 7, with three consecutive cardiac cycles are selected). Abbreviations: IVS;s and IVS;d, intraventricular septal wall thickness during systole and diastole; LVID;s and LVID;d, left ventricular internal diameter during systole and diastole; LVPW;s and LVPW;d, left ventricular posterior wall thickness during systole and diastole, IVRT, isovolumetric relaxation time, IVCT, isovolumetric contraction time; LV MPI, left ventricular myocardial performance index; AET, aortic ejection time.

Furthermore, echocardiography was employed to further evaluate potential structural or functional changes. No significant differences were observed in the area of the left atria between the WT and Het groups (Table 1C). In addition, the size and function of the left ventricle (LV) in WT and Het mice were assessed, with representative PLAX M-mode traces displayed in Fig. 3A. No significant changes were observed in the intraventricular septum (IVS) or LV internal diameter (LVID) at diastole or systole (Fig. 3B, C). However, there was a significant increase in the LV posterior wall thickness (LVPW) at both diastole and systole (Fig. 3D), indicating localised hypertrophy. Importantly, there were no significant differences in LV mass or volume at diastole and systole between Het mice and WT littermates, suggesting an absence of global hypertrophy (Table 1B).

Three parameters of systolic function were measured including ejection fraction (EF), fractional shortening (FS), and endocardial fractional area change (FAC), and none were found to be different in Het mice relative to WT mice (Table 1B, C). In addition, diastolic dysfunction, one of the earliest manifestations of HCM [18], was assessed by analysing pulse-wave doppler images of the mitral inflow. Isovolumetric relaxation time (IVRT) showed a trend towards an increase in Het mice compared to WT age-matched littermates (Fig. 3F). Isovolumetric contraction time (IVCT) was significantly elevated in Het hearts relative to WT hearts (Fig. 3G). The aortic ejection time (AET) was similar between the two genotypes (Fig. 3H). When evaluating global LV function using the LV myocardial performance index (LV MPI), there was a small but significant increase in Het mice compared to WT (Fig. 3I). Therefore, these findings indicate that while there were signs of subclinical changes in diastolic function, no overt diastolic dysfunction was detected.

### 3.4 Acnt2 M288T Het mice display no overt changes using strain analysis

Strain analysis is frequently used in clinical practice to identify early indications of heart dysfunction [19]. In this study, none of the strain parameters measured including radial and longitudinal strain and strain rate, exhibited significant differences between Het and WT controls (Table 2A). Furthermore, reverse strain analysis, a measure of diastolic function, indicated no substantial alterations in the Het mice, with the exception of a significant decrease in reverse radial strain (Table 2B). This finding confirms the presence of subclinical diastolic dysfunction in these mice, despite their overall well-preserved cardiac function.

**Table 2:** Two-dimensional speckle tracking showing strain, strain rate, and time-to-peak analysis of wildtype (WT) and *Actn2* M228T heterozygous (Het) at six months of age. (A) Strain and strain rate analysis in radial and longitudinal myocardial orientations showing no significant differences. (B) Reverse strain and strain rate analysis show no significant changes except a decrease in reverse radial strain in Het mice. (C) Time-to-peak (TTP) and TTP standard deviation (SD) analysis in radial and longitudinal orientations show no significant differences. (D) Reverse TTP and TTP SD showing no significant differences. Unpaired *t*-tests were used as the data was normally distributed except for radial and longitudinal strain TTP SD where the Mann-Whitney test was used. Data are expressed as mean ± SD.

Parameter	WT (*n* = 14)	Het (*n* = 13)	P-value
A) Strain analysis			
HR (bpm)	434 ± 44	429 ± 40	0.76
Radial strain (%)	26 ± 3.8	27 ± 7.8	0.58
Radial strain rate (1/s)	7.6 ± 1.5	8.2 ± 1.9	0.32
Longitudinal strain (%)	−12.7 ± 3.3	−12.7 ± 3.9	0.25
Longitudinal strain rate (1/s)	−4.7 ± 1.3	−4.9 ± 1.6	0.69

B) Reverse strain analysis			
Reverse radial strain (%)	−2.1 ± 1.1	−3.4 ± 1.7	⁎**0.012**
Reverse radial strain rate (1/s)	−8.4 ± 1.4	−9.4 ± 2.6	0.11
Reverse longitudinal strain (%)	1.6 ± 1.3	1.4 ± 0.6	0.55
Reverse longitudinal strain rate (1/s)	6.3 ± 2.1	5.7 ± 1.4	0.36

C) TTP analysis			
Radial strain TTP (ms)	58.6 ± 6.9	65.2 ± 11.5	0.07
Radial strain rate TTP SD (ms)	8.3 ± 6.3	15.4 ± 11.7	0.08
Longitudinal strain TTP (ms)	61.1 ± 6.8	59.9 ± 16.0	0.85
Longitudinal strain rate TTP SD (ms)	10.2 ± 9.3	13.2 ± 7.0	0.09

D) Reverse TTP analysis			
Reverse radial strain TTP (ms)	81.2 ± 24	82.5 ± 19	0.74
Reverse radial strain rate TTP SD (ms)	48.2 ± 13	56.1 ± 19	0.28
Reverse longitudinal strain TTP (ms)	83.1 ± 44	82.7 ± 39	0.67
Reverse longitudinal strain rate TTP SD (ms)	33.2 ± 21	40.8 ± 26	0.42

⁎ *p* < 0.05.

**Fig. 4:**
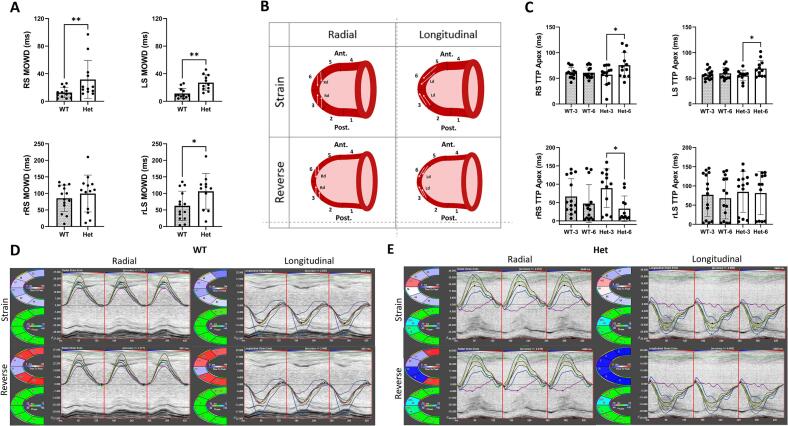
*Actn2* M228T Heterozygous (Het) mice show early signs of left ventricular dyssynchrony at six months of age when compared to their wild-type (WT) littermates. (A) Analysis of MOWD demonstrating significant alterations in radial and longitudinal directions. (B) Schematic representation of the left ventricular six anatomical segments, delineated as follows: segments 1 and 4: posterior and anterior base; segments 2 and 5: posterior and anterior middle; segments 3 and 6: posterior and anterior apex, respectively. The upper panel illustrates myocardial thickening and lengthening at radial and longitudinal strain analysis, respectively; the lower panel shows myocardial thinning and shortening at reverse radial and longitudinal strain analysis, respectively. (C) Analysis of time to peak (TTP) within segments 3 and 6 reveals dyssnchrony in Het group. (D, E) Representative images of strain and reverse strain analysis for both radial and longitudinal myocardial orientations in WT and Het groups, respectively. The majority of the data were normally distributed and unpaired T-tests were used. The Mann-Whitney test was employed for the non-normally distributed data (RS MOWD and LS MOWD). A One-way ANOVA was used for normally distributed data for comparisons among groups of two or more and the mean within the same group. A Kruskal-Wallis test was used for non-normally distributed data (rRS and rLS TTP Apex). Data are presented as mean ± S.D., *p < 0.05, **p < 0.01. (WT, n = 14; Het, n = 13). Abbreviations: Ant, anterior; Post, posterior; RS, radial strain; LS, longitudinal strain; rRS, reverse radial strain; rLS, reverse longitudinal strain; MOWD, maximum opposing wall delay; TTP, time to peak.

The analysis of time-to-peak (TTP) revealed no differences in the duration required for individual myocardial segments to achieve their respective peak strains. Additionally, peak strain dispersion and synchronicity, quantified by the standard deviation of TTP values (TTP SD), showed no significant changes in Het animals compared to WT for both radial and longitudinal analyses (Table 2C). A similar reverse TTP analysis also indicated no significant differences between the two groups (Table 2D). Further analysis of synchronicity using the maximum opposing wall delay (MOWD), revealed a significant delay in Het mice compared with WT controls (Fig. 4A). More specifically, the opposing apical segments (represented by segments 3 and 6 in Fig. 4B) exhibited dyssynchrony in the Het mice only, characterised by consistent differences in systolic radial and longitudinal TTP. In contrast, all other LV opposing segments demonstrated no discernible changes (Table S2).

## 4 Discussion

Our investigation revealed that *Actn2* M228T Het mice display atrial electrical alterations, as evidenced by increased APD and depolarisation time, without any accompanying changes in CV and no changes in ECG atrial electrophysiology. Importantly, these changes appear to be independent of any underlying structural or functional abnormalities, as the hearts of *Actn2* M228T Het mice exhibited normal cardiac structure and contractile function, with only subclinical changes indicative of hypertrophy and diastolic dysfunction. This mimics the situation of four variant carriers (out of 11 in total) in the original family with the pathogenic *ACTN2* M228T missense variant. These four individuals showed signs of atrial electrical remodelling in the absence of overt cardiac structural remodelling [7].

In our study, electrical abnormalities associated with the M228T variant were assessed in male and female six-month-old Het and WT mice, observing no discernible changes between the two sexes. This aligns with the observation that both male and female patients in the identified family exhibited similar phenotypes [7].

Interestingly, APD measured at 30, 50, and 70 % repolarisation showed significant or a trend towards prolongation in the Het atria. Prolongation of the APD is a common characteristic of HCM [20]. Furthermore, increase in APD can predispose individuals to arrhythmias [21] and has also been linked to an increased frequency of early afterdepolarisation (EADs) [22]. EADs are spontaneous depolarisations that occur during the plateau phase of action potential [23] and can also trigger new APs, leading to ectopic activity [24,25]. Therefore, the observed prolongation of APD in the mouse atria may indicate an increased susceptibility to ectopic activity and potential arrhythmogenic mechanisms [21] that underlie the atrial arrhythmias noted in the *ACTN2* M228T family [7]. In addition, a prolonged APD has been demonstrated to contribute to arrhythmia *via* several mechanisms which include an increased incident of repolarisation heterogeneity contributing to conduction block [26]. A prolonged APD has also been observed in several mouse models of AFib [27,28]. The prolongation of APD, however, was not accompanied by an increase in cardiac alternans, a common precursor to fibrillation [29].

Further studies are required to assess the ionic mechanism behind the observed changes in action potential morphology. Interestingly, prolonged depolarisation time was not reflected in a change in dF/dt, however ourselves and others have shown how upstroke morphology can be highly influenced by conduction dynamics, and there is a non-linear relationship between dF/dt and depolarisation time [13].

Analysis of the CV, derived from the comparison of activation times between different pixels of the activation map [30], showed no changes in either the left or right atria. This finding indicates that Het mice exhibit no alterations in activation time across the myocardial tissue, which is consistent with the ECG results that demonstrated no change in P-duration representing atrial conduction in conscious mice. Furthermore, the lack of change in CV, despite prolonged depolarisation time, is intriguing because prolongation of AP depolarisation is often concomitant with CV slowing due to a reduction in the inward sodium current [13,31]. Further studies are warranted to elucidate these mechanisms at the ionic level, including measurements of specific currents and calcium optical mapping to measure any alteration in cellular calcium.

A key question arises regarding whether the observed electrical changes stem from underlying structural and functional alterations in the *Actn2* M228T hearts. Our qPCR analysis of fibrosis and hypertrophy markers indicated no differences between genotypes, suggesting absence of structural remodelling. This was further confirmed using WGA staining, which showed no cellular hypertrophy in the atria of Het mice. This suggests that the atrial electrophysiological changes observed in the Het mice are likely to be independent of atrial structural remodelling or fibrosis.

Interestingly, echocardiography analysis of the ventricles revealed increased LVPW thickness in Het mice relative to age-matched WT mice, which might suggest early indications of a localised hypertrophic response. Previously, LVPW thickness has been recognised as an important indicator of the severity of LV hypertrophy [32]. As with clinical HCM, systolic function appeared normal, as indicated by preserved EF, FS, and FAC [33].

When evaluating diastolic function using cardiac time intervals, IVCT was significantly prolonged in Het mice. Previous studies have suggested that IVCT serves as an independent prognostic factor for atrial fibrillation [34] and is associated with an increased risk of heart failure [35]. In addition, the LV MPI, an indicator of global myocardial dysfunction [36,37], was significantly elevated in Het mice. Collectively, these observations reveal the absence of an overt HCM, yet suggest subclincal signs indicative of emerging HCM features. These findings align with the previously identified family study, where four members exhibited electrical pathologies without overt hypertrophy [7].

Strain analysis has emerged as a pivotal tool for evaluating cardiac function, clinically distiguishing early signs of dysfunction [38]. Although there were no changes in radial and longitudinal strain and strain rates in this study, signs of dyssynchrony were present in the Het mice. Increased MOWD, particularly the increased TTP between opposing apical segments in the Het group, indicated intraventricular dyssynchrony in specific cardiac segments, undetected by conventional echocardiography. The electrical alterations in the atria could have downstream effects on ventricular mechano-function, as patients with atrial fibrillation have been found to exhibit intraventricular dyssynchrony [39,40]. More recently, LV dyssynchrony has been shown as a predictor for AFib in a low-risk general population [41], and therefore this finding warrants further investigation.

The use of mouse models to study cardiac phenotypes, particularly HCM, closely related to human phenotypes often has limitations. Mouse models do not reflect all aspects of human disease. Prominent examples include previously generated knock-in HCM mouse models for *MYBPC3* [42] and *CSRP3* variants [43]. Fundamental differences in gene expression, transcriptional regulation, and intracellular signaling contribute to the distinct HCM profiles observed between humans and mice [44]. Furthermore, general anesthesia is often used to carry out imaging protocols such as echocardiography in mice which results in cardiac and respiratory depression, affecting parameters analysed [45,46].

Additional challenges in assessing electrical phenotypes arise from the significant differences in the ECG characteristics between humans and mice. Mice typically exhibit higher heart rates (600–800 bpm) than humans, resulting in faster depolarisation and repolarisation phases, which leads to shorter ECG intervals and complicates direct comparisons [47]. Furthermore, mice do not develop spontaneous AFib, except under experimentally induced settings [48]. The experimental modelling of AFib in mice poses challenges, as it fails to capture the complex pathology of the disease [49]. These combined factors make the characterisation of electrophysiological phenotypes in mouse models challenging.

In summary, our investigation into the HCM-linked *ACTN2* M228T variant revealed Het mice display atrial electrical alterations, as evidenced by increased APD and depolarisation time, which may signify an arrhythmogenic phenotype that parallels the electro-pathological phenotype identified in the original family. Importantly, these changes appear to be independent of any underlying structural or functional abnormalities, as the hearts display only subclinical structural and functional changes indicative of HCM, with no structural remodelling being evident in the atria.

To fully characterise the *ACTN2* M228T variant, further studies using *in vitro* human cellular models are essential. Similar studies have been performed on another *ACTN2* variant, T247M [50]. However, the mechanisms by which *ACNT2* variants can lead to electrical dysfunction in the atria remain unexplored. Future work will employ human induced pluripotent stem cell–derived atrial cardiomyocytes (hiPSC-aCM), which will offer deeper insights into the electrophysiological impact of this variant at ionic level, complementing the findings from the current *in vivo* mouse model.

## CRediT authorship contribution statement

**Maya Noureddine:** Writing – review & editing, Writing – original draft, Methodology, Formal analysis, Conceptualization. **Sophie Broadway-Stringer:** Writing – review & editing, Writing – original draft, Methodology, Formal analysis, Conceptualization. **Christopher O'Shea:** Writing – review & editing, Formal analysis. **Bethany A.I. Jones:** Data curation, Writing – review & editing. **Abbie Hayes:** Data curation, Writing – review & editing. **Chris Denning:** Writing – review & editing, Supervision. **Siobhan Loughna:** Writing – review & editing, Supervision. **Fiyaz Mohammed:** Writing – review & editing, Supervision. **Davor Pavlovic:** Writing – review & editing. **Katja Gehmlich:** Writing – review & editing, Supervision, Project administration, Methodology, Formal analysis, Conceptualization.

## Declaration of competing interest

The authors declare the following financial interests/personal relationships which may be considered as potential competing interests: Given his role as Editor-in-Chief for JMCCPLUS journal, Davor Pavlovic had no involvement in the peer review of this article and had no access to information regarding its peer review. Full responsibility for the editorial process for this article was delegated to another journal editor.

## Appendix A Supplementary data

Supplementary material Image 1
