# Supplementary material for: Atrial electrical alterations with intact cardiac structure and contractile function in a mouse model of an HCM-linked ACTN2 variant
Source: J Mol Cell Cardiol Plus. 2025 May 17;12:100455. doi: 10.1016/j.jmccpl.2025.100455 (PMC12153375; doi:10.1016/j.jmccpl.2025.100455)
Supplement: Supplementary file 1 — Supplementary material [file mmc1.pdf]

Atrial Electrical Alterations with Intact Cardiac Structure and Contractile Function in a Mouse Model of an HCM-linked *ACTN2* Variant

Supplementary Material

Maya Noureddine[1‡], Sophie Broadway-Stringer[1‡], Christopher O'Shea[1,2], Bethany AI Jones[1], Abbie Hayes[1], Chris Denning[3], Siobhan Loughna[4], Fiyaz Mohammed[5], Davor Pavlovic[1], Katja Gehmlich[1,6*]

[1]Department of Cardiovascular Sciences, School of Medical Sciences, College of Medicine and Health, University of Birmingham, Birmingham, UK

[2]Division of Biomedical Sciences, Warwick Medical School, Clinical Sciences Research Laboratory, Coventry, UK

[3]Biodiscovery Institute, University of Nottingham, Nottingham, UK

[4]School of Life Sciences, Faculty of Medicine and Health Sciences, University of Nottingham, Nottingham, UK

[5]Department of Immunology and Immunotherapy, School of Infection, Inflammation and Immunology, College of Medicine and Health, University of Birmingham, Birmingham, UK

[6]Division of Cardiovascular Medicine, Radcliffe Department of Medicine and British Heart Foundation Centre of Research Excellence Oxford, University of Oxford, Oxford, UK

*Correspondence:
Katja Gehmlich: k.gehmlich@bham.ac.uk

‡M. Noureddine and S. Broadway-Stringer equally contributed to this work as first authors

1. Detailed Methodology:

1.1.Cardiac optical mapping on mouse atria

Prior to performing optical mapping on mouse atria, a two-litre stock solution of Krebs- Henseleit was prepared. This solution was filtered and equilibrated with 95% O_2 and 5% CO_2 for 5 mins. Mice were then placed in an anaesthesia induction chamber filled with 4% isoflurane and supplemented with 95% O_2 and 5% CO_2 at a rate of 2 L/min. Once pedal withdrawal reflex was absent, the mouse was positioned supine, and a scavenger mask supplied with 4% isoflurane and 95% O_2 was placed over the nose. The extremities were subsequently secured with tape. Heparin (0.1 ml of 1000 I.U./ml) was administered via intraperitoneal injection to prevent clot formation during heart collection. After one minute, the absence of pedal reflex test was re-evaluated to ensure appropriate anesthetic depth. The chest was then opened, and the heart with intact ascending aorta removed. In accordance with UK Home Office Schedule 1 regulations, cervical dislocation was performed to confirm death. The harvested hearts were transferred to a dish containing cold Krebs solution, and excess tissue and fat surrounding the heart and aorta were removed. The ascending aorta was cannulated and secured with a knot of thread and perfused with Krebs solution (previously cooled to 4 °C). The heart was mounted onto a vertical Langendorff apparatus. Retrograde perfusion of the heart was performed using Krebs-Henseleit solution, equilibrated with 95% O_2 and 5% CO_2 and heated to 37°C at a flow rate of 4-4.5 ml/min. Warm Krebs solution was mixed with a voltage-sensitive dye (Di-4-ANEPPS, 5 mg/ml, BIOTIUM), which was infused into the aorta via bolus injection for 3-5 minutes. Following perfusion, the heart was removed from the Langendorff apparatus and placed in a dish containing warm Krebs solution.

The left and right atria were dissected and pinned using an optical mapping rig. The optical mapping rig was positioned under the optical mapping cameras which were connected to a super-fusion system of warmed Krebs solution (37°C), equilibrated with 95% O_2 and 5% CO_2, and supplemented with 42.75 µM Blebbistatin (Med Chem Express). The tissues were left for 5-10 mins with Krebs solution to equilibrate and allow contraction to abate. For optical mapping recordings, four LEDs at 530/50 nm illuminated the atria and filtered fluorescence (>630 nm) was collected using ORCA flash 4.0 sCMOS camera. Images were collected at 500 Hz sampling rate with a maximal resolution of 200 × 2048 pixels (71 µm/pixel). The Win Fluor software (Fluorescence Image Capture & Analysis Program V4.1.9) was connected to the optical mapping

cameras and used to visualise the atria and assess spontaneous firing. A bipolar platinum stimulating electrode was positioned at the edge of the atrium. A pacing protocol was implemented using Spike2 software, with pacing at cycle lengths of 120, 100, and 80 ms for 100 stimuli (2ms pulse width, 2x diastolic threshold), incorporating a 5-second pause between pacing sequences, with 100 pulses delivered at each cycle length. The physiologically relevant heart rate (in beats per minute) was calculated using the following formula: Heart rate (bpm)=60000/cycle length (ms).

Data files were exported as TIFF files and converted to MAT format using Win Fluor software. ElectroMap software was employed to analyse the last 20 beats at each pacing cycle length, to allow for adaptation to the new pacing rate [1]. The region of interest (ROI) was defined as the border of the atria, and the parameters (described below) were measured at each pixel location (~1000 pixels/atria). Unless stated otherwise, the mean value of the parameters across the tissue was then calculated and used for grouped analysis. Prior to parameter measurement, preprocessing was applied using a Gaussian spatial filtering (3x3 pixel area) and top-hat baseline correction (100ms length).

Conduction velocity was determined using the multi-vector method [2], and data with a CV less than 20 cm/s was excluded from analysis. Action potential duration (APD) was assessed at 30%, 50%, and 70% repolarisation, measured from the time of maximum upstroke velocity (dF/dt). Triangulation was calculated by determining the difference between APD 70 and APD 30, as previously documented [3, 4]. Depolarisation time was measured between 10 and 90% upstroke amplitude. The analysis of upstroke (dF/dt) was also carried out using electromap as described [2]. Alternans analysis was assessed using the difference between the APD 70 in the last two beats at each cycle length.

1.2.Echocardiography

Mice were placed in an anaesthesia induction chamber filled with 4% isoflurane, supplemented with 100% O_2 at a flow rate of 2 L/min. Mice were transferred to a warming platform to maintain body temperature at 37°C. General anaesthesia was maintained using a scavenger mask delivering 1-2% isoflurane and 100% O_2 at a flow rate of 2 L/min. The mice were secured in a supine position, with their paws taped to electrode pads that had been pre-coated with electrode conduction gel.

Hair from the chest area was removed using depilatory cream (Nair), and the area was cleaned with warm water before applying warmed ultrasound gel. A Vevo F2 ultrasound system (Fujifilm VisualSonics Inc) was used to image the heart using a UHF57X transducer. To visualise the heart in a parasternal long-axis view (PLAX), the transducer was angled parallel to the sternum of the mouse and rotated slightly towards the right shoulder of the mouse. For short-axis view (SAX), the transducer was rotated 90-degrees clockwise. The left atrial area was measured using B-mode images in the PLAX view. The right atrial area was not assessed as apical four chamber view images were not recorded. Fractional area change (FAC) was assessed using B-mode images in the SAX view. Other echocardiography parameters were assessed using M-mode images in the PLAX view and pulse-wave Doppler imaging of mitral inflow. Measurements were quantified offline using Vevo Lab 5.7.1 (Fujifilm VisualSonics Inc). Data from three consecutive cardiac cycles were averaged and presented. Heart rate was maintained at 450 ± 50 bpm. Due to technical reasons, some measurements were excluded.

1.3. Speckle tracking echocardiography

Two-dimensional echocardiography loops (B-mode) previously recorded in the PLAX view were selected for speckle tracking and strain analysis using VevoLabs Strain 1.0 software (Fujifilm VisualSonics Inc). Cine loops with a frame rate of >223 FPS and clear endocardial borders were used. Strain analysis encompassed three consecutive cardiac cycles, excluding any respiratory variations. The software divided the left ventricular wall into six equal anatomical segments. Strain, strain rate, and Time-to-peak (TTP) in the radial and longitudinal myocardial orientations were calculated and averaged across the six segments. The standard deviation of TTP was calculated across the six segments. In addition, the software automatically measured the maximal opposing wall delay (MOWD) across all segments by calculating the maximum time lag that occurs between opposing segments. A reverse-peak tool was employed to assess the strain, strain rate, and TTP during diastole.

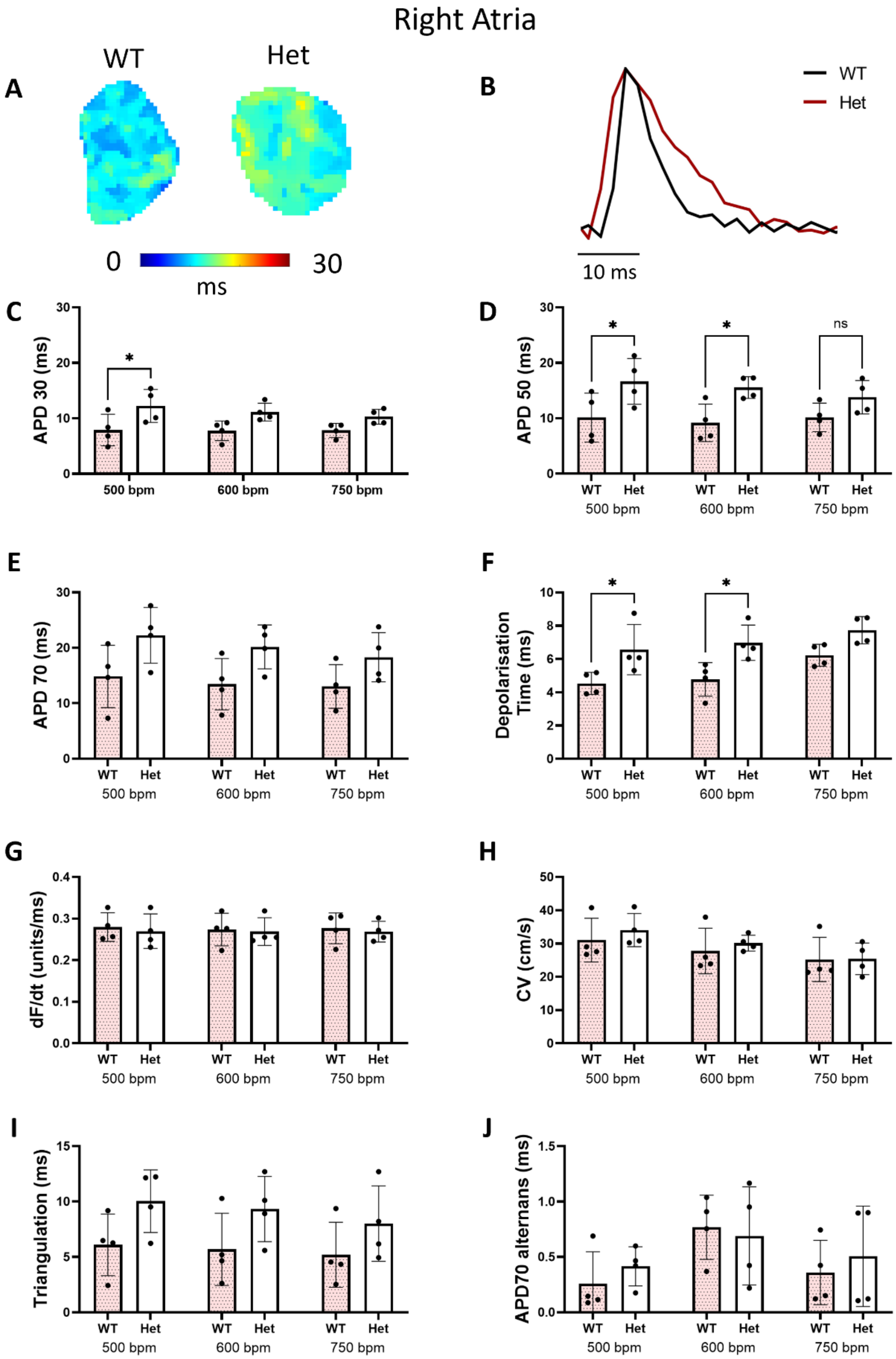
Right Atria
A
WT
Het
0
30
ms
B
WT
Het
10 ms
C
APD 30 (ms)
*
500 bpm
600 bpm
750 bpm
D
APD 50 (ms)
*
*
ns
WT
Het
500 bpm
600 bpm
750 bpm
E
APD 70 (ms)
F
Depolarisation Time (ms)
G
dF/dt (units/ms)
H
CV (cm/s)
I
Triangulation (ms)
J
APD70 alternans (ms)

Figure S1: Optical mapping analysis on mice right atria of wildtype (WT) and *Actn2* M228T heterozygous (Het) at six months of age showing atrial electrical alterations. (A) Representative heat maps at 50% of repolarisation at 500 bpm for the right atria. (B) Representative action potential traces of right atria showing a delay in Het mice. (C, D, E) Analysis of action potential duration (APD) at 30, 50 and 70% repolarisation showing an increase in Het mice. (F) Depolarisation time analysis of right atria shows increase in Het mice. (G) No significant changes in upstroke (dF/dt) in Het mice. (H, I, J) Conduction velocity (CV), triangulation, and alternans measurements show no significant changes in right atria of Het mice. Two-way ANOVA was used to analyse data. Values are presented as mean ± SD * $p < 0.05$;. (WT, n=4; Het, n=4).

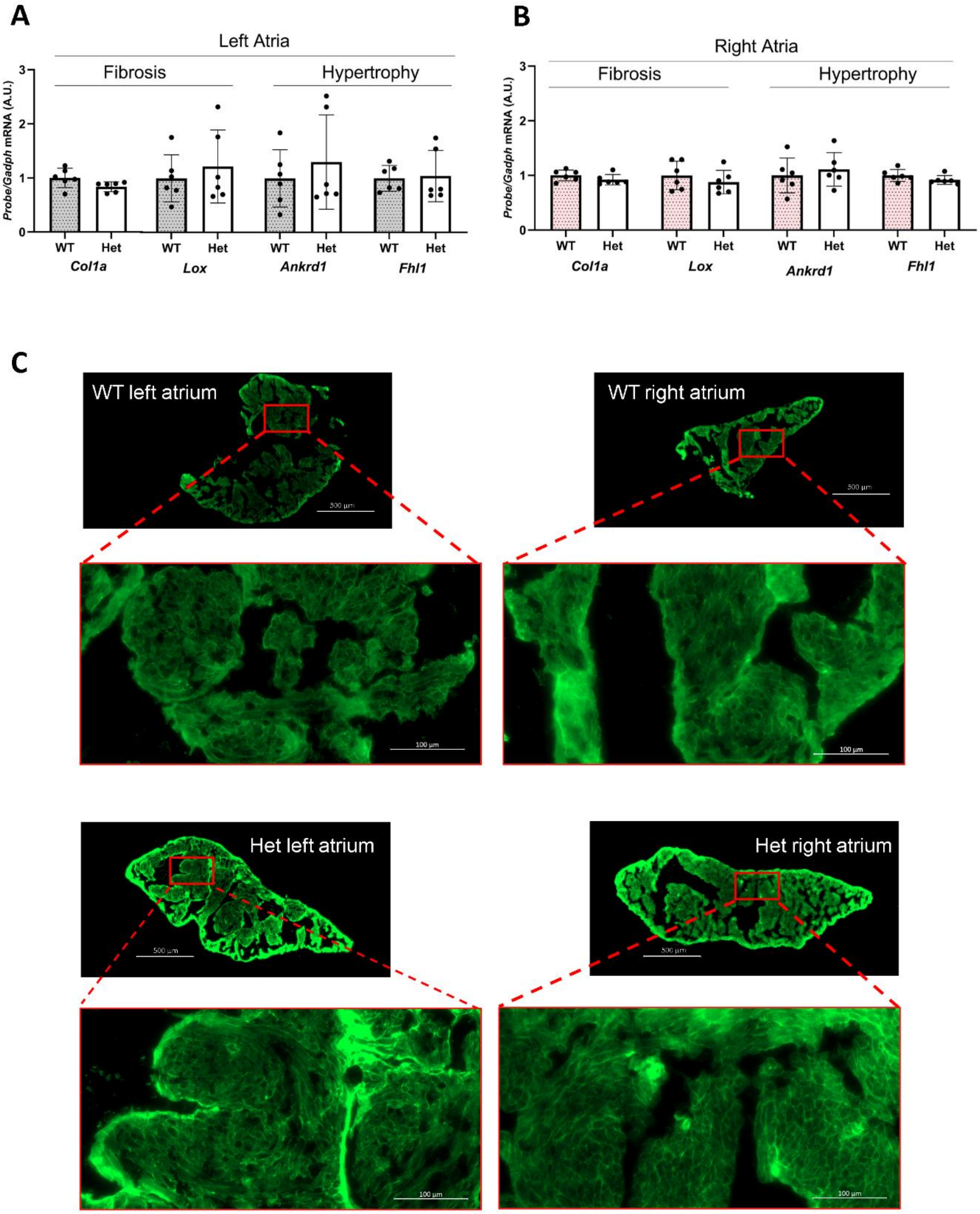

A
Left Atria
Fibrosis
Hypertrophy
Probe/Gadph mRNA (A.U.)
WT
Het
Col1a
Lox
Ankrd1
Fhl1
B
Right Atria
Fibrosis
Hypertrophy
Probe/Gadph mRNA (A.U.)
WT
Het
Col1a
Lox
Ankrd1
Fhl1
C
WT left atrium
WT right atrium
Het left atrium
Het right atrium

Figure S2: qPCR and histology analysis of the right and left atria of wildtype (WT) and *Actn2* M228T heterozygous (Het) at six months of age showing no evidence of atrial structural remodelling. (A) qPCR analysis of fibrosis markers (*Col1a* and *Lox*), and hypertrophy markers (*Ankrd1* and *Fhl1*) showing no changes in the left atria. (B) qPCR analysis of fibrosis and hypertrophy markers in the right atria showing no difference between WT and Het mice. Student's t-test was used as the data was normally distributed except for *Col1a* (in left and right atria), and *Ankrd1* and *Fhl1* in left atria, where the Mann-Whitney test was used. Values are presented as mean ± SD, (WT, n=6; Het, n=6 as biological replicates = atrial samples from individual mice; qPCRs were run in triplicates = technical replicates). (C) WGA staining of left and right atrial cryo-sections. In enlarged areas (red boxes) cell outlines showed no change of cell size between WT and Het mice. Two biological replicates (= atrial samples from individual mice), with one representative shown. Two technical replicates (= sections stained per atrium, with at least two areas imaged).

Transcript	Species	Assay ID	Label
Col1a	mouse	Mm00801666_g1	FAM-MGB
Lox	mouse	Mm00495386_m1	FAM-MGB
Ankrd1	mouse	Mm00496512_m1	FAM-MGB
Fhl1	mouse	Mm04204611_g1	FAM-MGB
Gadph	mouse	4352339E	VIC-MGB

Table S1: Table summarising TaqMan assays (Applied Biosystems) used for qPCR.

Parameter	WT-1	WT-4	P-value (WT)	Het-1	Het-4	P-value (Het)
**A) Base Segment**	**Post.**	**Ant.**		**Post.**	**Ant.**	
Radial Strain TTP (ms)	52 ± 16	57 ± 9	0.99	65 ± 14	58 ± 11	0.99
Longitudinal Strain TTP (ms)	66 ± 11	62 ± 19	0.94	61 ± 15	51 ± 18	0.99
Reverse Radial Strain TTP (ms)	110 ± 29	118 ± 13	0.99	114 ± 32	130 ± 10	0.99
Reverse Longitudinal Strain TTP (ms)	112 ± 29	113 ± 27	0.97	112 ± 50	124 ± 46	0.91
**Parameter**	**WT-2**	**WT-5**	**WT P-value**	**Het-2**	**Het-5**	**Het P-value**
**B) Middle Segment**	**Post.**	**Ant.**		**Post.**	**Ant.**	
Radial Strain TTP (ms)	61 ± 10	59 ± 9	0.99	62 ± 8	59 ± 8	0.99
Longitudinal Strain TTP (ms)	64 ± 10	63 ± 18	0.99	62 ± 16	57 ± 10	0.99
Reverse Radial Strain TTP (ms)	111 ± 39	118 ± 26	0.99	109 ± 30	110 ± 29	0.99
Reverse Longitudinal Strain TTP (ms)	117 ± 32	115 ± 29	0.99	119 ± 25	129 ± 39	0.99

Table S2: Analysis of time to peak (TTP) of the base and middle opposing segments in radial and longitudinal myocardial orientations showing no significant differences between genotypes. (A) No significant changes were observed when comparing base opposing segments within the same group of TTP strain in longitudinal and radial orientations (segment 1: posterior base, segment 4: anterior base). (B) No significant changes present in the middle opposing segments within the same group of TTP strain in longitudinal and radial orientations (segment 2: posterior middle, segment 5: anterior middle).One-way ANOVA was used for normally distributed data (reverse longitudinal strain TTP in the base segment), and the mean within the same group was compared and reported. The Kruskal-Wallis test was used on the rest of the data sets as they were not normally distributed. Values are presented as mean ± S.D. (WT, n=14; Het, n=13).

References:

[1] C. O'Shea, A.P. Holmes, T.Y. Yu, J. Winter, S.P. Wells, B.A. Parker, D. Fobian, D.M. Johnson, J. Correia, P. Kirchhof, L. Fabritz, K. Rajpoot, D. Pavlovic, High-Throughput Analysis of Optical Mapping Data Using ElectroMap, J Vis Exp (148) (2019).

[2] C. O'Shea, D. Pavlovic, K. Rajpoot, J. Winter, Examination of the Effects of Conduction Slowing on the Upstroke of Optically Recorded Action Potentials, Front Physiol 10 (2019) 1295.

[3] T. Kubo, T. Ashihara, T. Tsubouchi, M. Horie, Significance of integrated in silico transmural ventricular wedge preparation models of human non-failing and failing hearts for safety evaluation of drug candidates, J Pharmacol Toxicol Methods 83 (2017) 30-41.

[4] Y. Qu, G. Page, N. Abi-Gerges, P.E. Miller, A. Ghetti, H.M. Vargas, Action Potential Recording and Pro-arrhythmia Risk Analysis in Human Ventricular Trabeculae, Front Physiol 8 (2017) 1109.
